# One-Year Adherence to Continuous Positive Airway Pressure With Telemonitoring in Sleep Apnea Hypopnea Syndrome: A Randomized Controlled Trial

**DOI:** 10.3389/fmed.2021.626361

**Published:** 2021-04-20

**Authors:** Olivier Contal, William Poncin, Stéphanie Vaudan, Angélique De Lys, Hiromitsu Takahashi, Séverine Bochet, Stéphane Grandin, Philippe Kehrer, Florian Charbonnier

**Affiliations:** ^1^School of Health Sciences Haute Ecole de Santé Vaud (HESAV), Haute école spécialisée de Suisse occidentale (HES-SO) University of Applied Sciences and Arts Western Switzerland, Delémont, Switzerland; ^2^Institut de recherche expérimentale et clinique (IREC), pôle de Pneumologie, oto-rhino-laryngologie (ORL) et Dermatologie, Université Catholique de Louvain, Brussels, Belgium; ^3^Geneva Pulmonary League, Geneva, Switzerland; ^4^Hôpital du Valais, Service de Physiothérapie, Martigny, Switzerland; ^5^Centre de Médecine du Sommeil et de L'éveil, Geneva, Switzerland; ^6^Service de Pneumologie, Département des Spécialités de Médecine, Hôpitaux Universitaires Genevois (HUG), Geneva, Switzerland

**Keywords:** telemedicine, CPAP, obstructive sleep apnea, adherence, health care resources

## Abstract

**Study Objective:** Telemedicine (TM) for continuous positive airway pressure (CPAP) treated patients may save health-care resources without compromising treatment effectiveness. We assessed the effect of TM (AirView Online System, ResMed) during the CPAP habituation phase on 3-month and 1-year treatment adherence and efficacy in patients with moderate-to-severe obstructive sleep apnea (OSA).

**Methods:** At CPAP initiation, 120 patients diagnosed with OSA were randomized to either usual care (UC) or TM during the habituation phase (clinical registration: ISRCTN12865936). Both groups received a first face-to-face appointment with a sleep care giver at CPAP initiation. Within the following month, 2 other physical visits were scheduled in the UC group whereas two phone consultations were planned in the TM group, in which CPAP parameters were remotely adapted. Additional physical visits were programmed at the patient's request. Face-to-face consultations were scheduled at 3 and 12 months after CPAP initiation. The primary outcome was the mean CPAP daily use over the course of 12 months.

**Results:** Twenty of 60 patients stopped CPAP therapy in the UC group vs. 14 of 60 in the TM group (*p* = 0.24). In per protocol analysis, mean [95% CI] daily CPAP use among 86 patients still using CPAP at 12 months was 279 [237; 321] min in the 38 patients on UC and 279 [247; 311] min in the 43 patients on TM, mean difference [95% CI]: 0 [−52; 52] min, *P* = 0.99. Total consultation time per patient was not different between groups, TM: 163 [147; 178] min, UC: 178 [159; 197] min, difference: −15 [−39; 9] min, *p* = 0.22.

**Conclusions:** Telemedicine during the CPAP habituation phase did not alter daily CPAP use or treatment adherence and did not require more healthcare time. Telemedicine may support clinic attendance for CPAP titration.

**Clinical Trial Registration:** [ISRCTN], identifier [ISRCTN12865936].

## Introduction

Obstructive sleep apnea (OSA) is a common sleep disorder with an estimated prevalence of 19% in the general population (1). Untreated, OSA is an important cause of morbidity and mortality (2). Continuous positive airway pressure (CPAP), the first-line treatment option offered to patients with OSA (3), has proven to improve sleep quality (4), to decrease impaired sleep-related morbidities (5–7), and to reduce the risk of traffic accidents (8, 9). However, adequate adherence to this treatment, defined as use for at least 4 h per night over more than 70% of nights (10), remains a challenge for patients with OSA. It is estimated that a quarter to half of patients do not achieve adequate adherence (11–13). Among factors that have been associated with poor long-term adherence, low CPAP use and side-effects during the beginning of the treatment have been identified as essential factors (14).

Therapeutic strategies aiming at improving the early experience of CPAP treatment are therefore relevant. Telemedicine (TM), which refers to the exchange of patient data with the purpose of enhancing disease management (15), might be an attractive option since it has the potential to promptly address treatment-related problems without further increasing the workload of sleep specialist consultation. Indeed, studies in recent years have shown that CPAP telemonitoring (i.e., wireless transmission of physiological or non-invasive data) can optimize the management of patients with OSA by saving the nursing time without affecting patient's adherence or satisfaction (15). However, telemonitoring strategies often imply regular transmission of data and check-ups by a qualified caregiver (16–18). This healthcare organization may not be applicable in all countries or sleep centers (19). Furthermore, in some locations such as in Geneva (Switzerland), the standard CPAP habituation phase of 1 month includes three visits or even more at therapist's demand or patient's request, thereby already offering a rapid intervention to any troubles experienced by the patient. The role of TM in this context is unclear. Therefore, we aimed to verify if a TM intervention during the critical habituation phase, consisting in replacing face-to-face visits by telephonic consultations with remote CPAP parameters adaptation, can maintain the short- and long-term effectiveness of CPAP treatment.

The purpose of this study was to evaluate the impact of telemedicine during the habituation phase on treatment adherence and efficacy at 3 months and 1 year of follow-up.

## Methods

### Participants

Patients were referred to the trial by three primary care pulmonologists in Geneva. As soon as the diagnosis of OSA and the prescription of CPAP were made, eligible participants were proposed to enter the study. Patients aged at least 18 years or older with an apnoea–hypopnoea index (AHI) of more than 15 events per hour were deemed eligible. Exclusion criteria were prior exposure to a treatment for OSA, language barriers, and any disorders or behavioral difficulties likely to hamper adequate cooperation or comprehension regarding CPAP therapy. All included patients provided written informed consent to participate in the study. The study protocol was approved by the cantonal research ethics committee of Geneva (CCER) and is registered on ISRCTN database (ISRCTN12865936).

### Study Design

Before starting pressure titration, participants were randomized to have CPAP therapy managed during the habituation phase with face-to-face consultations (usual care - UC group) or *via* teleconsultation (TM group). Once a patient has consented to enter the trial an opaque envelope is opened and the patient is then offered the allocated treatment regimen. All patients were treated with the CPAP device AirSense 10 (Resmed).

According to our standard practice, CPAP initiation and habituation phases typically extend over a period of 1 month and include three appointments or more with a sleep care givers. During the first appointment, education about the disease and the CPAP device is provided, the adequate interface is identified, and the way to set the CPAP for titration with autoadjusting (APAP) mode is explained. This session was kept unchanged in both UC and TM groups. The following data were recorded during this visit: age, gender, body mass index, subjective daytime sleepiness measured by the Epworth Sleepiness Scale (ESS) (20), and depression measured by the QD2A scale (21, 22). The two next appointments took place 1 or 2 weeks after the previous ones and differed between groups. In the UC group, conventional face-to-face consultations were scheduled in which CPAP treatment information (pressure, leaks, residual respiratory events, and adherence) were downloaded, management of side-effects was discussed, and treatment pressure was fixed at the 95th percentile pressure during the titration phrase. In the TM group, those two later consultations took place *via* phone calls. The sleep care givers remotely downloaded CPAP treatment information through the airview program, reviewed them with the patient and discussed the management of side effects by phone. Treatment pressure was also remotely fixed *via* airview. Additional physical visits were programmed at therapist's judgment (if the sleep care givers deemed it necessary to further improve the quality of treatment) or at the patient's request.

Afterward, all patients were scheduled for follow-up physical appointments with their pulmonologist 3 and 12 months after the beginning of CPAP therapy. CPAP usage data were collected and clinical evaluation (ESS and QD2A) was performed. The proportion of patients continuing with their treatment was also recorded.

Adherence was measured as a proportion of patients continuing their CPAP therapy at the end of the trial and as the average time of CPAP usage over 1 year (primary outcome) and 3 months. Care time [i.e., time spent to each consultation, to administrative procedures and to the airview program (TM only)] was registered by the sleep care givers in charge of the patient.

The overall cost of each intervention was estimated taking into account the hourly cost related to the salary of the sleep care givers, the rental of the office consultation room and patient's cost related to travel to the consultation location. The salary of the sleep care givers (50 CHF/h) and the office room fees (25 CHF/consultation) were provided by the Geneva Lung Association. The patient's traveling cost was estimated based on two bus tickets (roundtrip) per visit (6 CHF/visit) considering that bus is the most used way of traveling in Geneva, due to the city's compact size and good public transport facilities.

### Statistical Analysis

The primary outcome was the mean CPAP daily use over the course of 12 months. Assuming an α risk of 0.05 and a β risk of 0.2 in a two-sided test, a sample size of 57 subjects in each group was needed to detect a difference of 60 min in mean daily use of CPAP with a standard deviation between the two groups of 115 min (4 and 5 h of daily use in TM and UC group, respectively) (18). To allow for 5% drop out, we aimed to recruit 60 patients in each group.

Normality of data was verified with Kolmogorov-Smirnov and Shapiro-Wilk tests. Data are presented as means [95% CI] or as frequency in percentages. Between-groups comparisons were performed by independent Student *t*-tests for continuous variables of chi-square tests for categorical variables. A two-way mixed analysis of variance with time (baseline, 3-month, 12-month) as within-subjects factor and group (UC vs. TM) as the between-subjects factor was conducted. We verified the equality of variance using the Mauchley's test of sphericity. If sphericity was violated, the Greenhouse-Geisser correction was used. Bonferroni method was used for *post-hoc* comparisons. The time at CPAP withdrawal was compared between groups using Kaplan-Meier plot analysis. A regression analysis was conducted to verify if sex or age were predictors of CPAP adherence at 12 months.

Missing data were not replaced, and the primary outcome was assessed among all patients still using CPAP therapy after 12 months. All tests were two-sided and *p*-values < 0.05 were considered statistically significant. Statistical analyses were performed using Stata version 15 (StataCorp, College Station, TX, USA).

## Results

**Figure 1:**
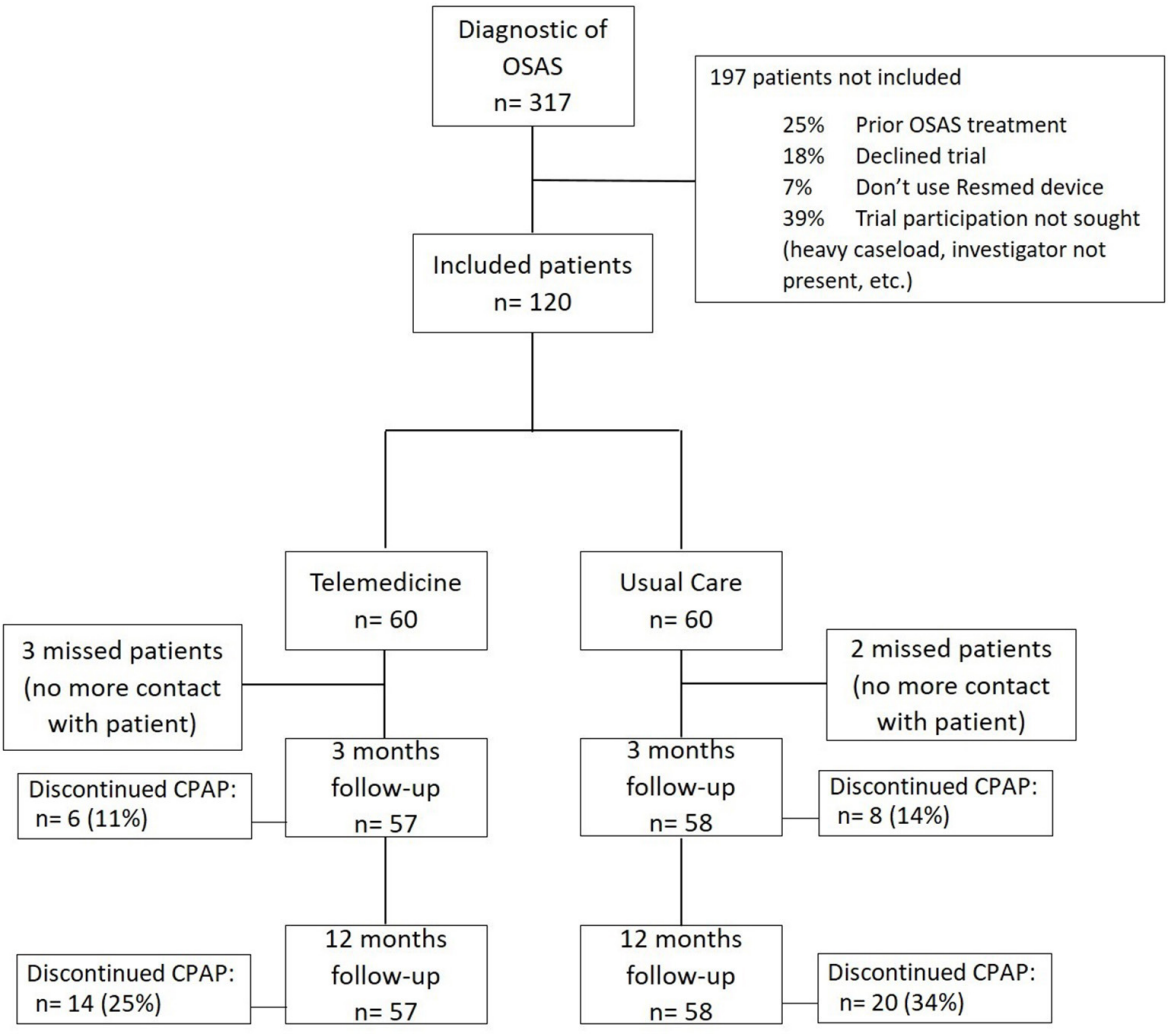
Study flow chart.

A total of 120 patients were included and randomized to the UC or TM group. During the first 3-month period of CPAP therapy, three patients were lost to follow-up in the UC group and two patients in the TM group (Figure 1). Patient characteristics are shown in Table 1. Final analyses were performed on data for the remaining 115 patients.

**Table 1:** Baseline characteristics.

	**UC group**	**TM group**	***p*-value**
	**(*n* = 60)**	**(*n* = 60)**	
Age (years)	57 [54; 60]	54 [48; 59]	0.29
Males (%)	39 (65%)	40 (67%)	0.99
BMI (kg/m^2^)	32 [30; 34]	31 [29; 33]	0.36
AHI (events/h)	43 [38; 49]	37 [32; 42]	0.09
ESS score	10.2 [9; 12]	10.3 [9; 12]	0.97
Depression score	4.3 [3.1; 5.4]	4.4 [3.4; 5.4]	0.83

*Data are displayed as means [95% CI] or proportions. Depression score was calculated using the QD2A questionnaire*.

*AHI, apnoea–hypopnoea index; BMI, body mass index; ESS, Epworth Sleepiness Scale; TM, telemedicine; UC, usual care*.

### Treatment Effectiveness

**Figure 2:**
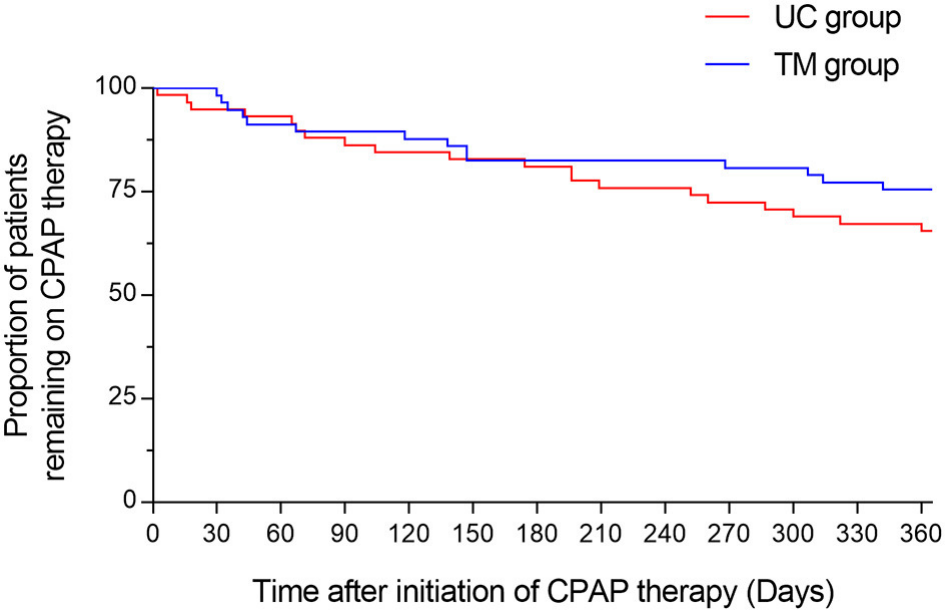
Kaplan-Meier curve of patients abandoning their CPAP therapy. The figure displays the percentage of participants who remain treated with their CPAP during a period of 1 year. The red line refers to the participants randomized in the usual care (UC) group, the blue line refers to the participants randomized in the telemonitoring (TM) group.

Treatment adherence in terms of daily use of CPAP therapy was similar between groups at 3 months and 1 year (Table 2). Likewise, the proportion of patients discontinuing CPAP therapy did not differ between groups at any time points (Figure 2). Sex or age were not predictors for CPAP adherence. Out of the remaining 79 patients continuing their CPAP therapy at the end of the study period, 50% (19/38) and 51% (22/43) of patients in the UC and TM groups, respectively were considered to be adherent (i.e., average CPAP use for at least 4 h per night over more than 70% of nights) at 1 year. Twenty-six patients out of 60 in the TM group needed a physical appointment. The consultations were mostly for mask problems. The groups did not differ in terms of therapeutic CPAP pressure, residual AHI, or mask leak (Table 2). No adverse events were noted during the study.

**Table 2:** Comparison of adherence, CPAP parameters, healthcare time, and overall costs between both groups.

	**UC group** **(*n* = 58)**	**TM group** **(*n* = 57)**	**Mean difference [95% CI], *p*-value**
**Adherence**			
Mean daily use of CPAP (min)			
Over the course of the first 3 months	262 [220; 305] (*n* = 50)^*^	288 [257; 319] (*n* = 51)^*^	−25 [−77; 26], *p* = 0.34
Over the course of the first 12 months	279 [237; 321] (*n* = 38)^*^	279 [247; 311] (*n* = 43)^*^	0 [−52; 52], 0.99
Proportion of patients discontinuing CPAP (*n*, %)			
At 3 months	8 (14%)	6 (11%)	*p* = 0.59
At 12 months	20 (34%)	14 (25%)	*p* = 0.24
**CPAP parameters at 1 year**			
CPAP pressure (cmH_2_O)	11.4 [10.7; 12.1]	11.0 [10.4; 11.6]	0.4 [−0.5; 1.3], *p* = 0.35
Residual AHI (events/h)	1.8 [1.0; 2.5]	1.7 [1.3; 2.1]	0.1 [−0.7; 0.9], *p* = 0.78
Leaks at 95th percentile (L/min)	26 [20; 32]	24 [20; 27]	2 [−4; 8], *p* = 0.50
**Healthcare time**			
Total consultation time (min)	163 [147; 178]	178 [159; 197]	−15 [−39; 9], *p* = 0.22
Administrative time (min)	50 [45; 57]	56 [50; 62]	−5 [−14; 3], *p* = 0.23
Airview management time (min)	NA	22 [19; 25]	NA
Education-management time (min)	112 [101; 123]	99 [88; 112]	12 [−4; 27], *p* = 0.13
Mean time per consultation (min)	33 [31; 34]	29 [28; 31]	3 [1; 6], *p* = 0.004
Total consultation number (*n*)	5 [4.6; 5.5]	6.3 [5.6; 6.9]	−1.2 [−2.1; −0.4], *p* = 0.003
Face-to-face consultation number (*n*)	4.8 [4.4; 5.3]	3.2 [2.9; 3.6]	1.6 [1.0; 2.2], *p* <0.001
**Average cost (CHF) per patient**			
Total	285 [259; 312]	248 [222; 274]	37 [0; 73], *p* = 0.047
Sleep care givers salary	136 [123; 149]	148 [132; 164]	−12 [−33; 8], *p* = 0.22
Office room rental	121 [123; 149]	81 [71; 90]	40 [25; 55], *p* <0.001
Public transportation	29 [26; 32]	19 [17; 22]	10 [6; 13], *p* <0.001

*Data are displayed as means [95% CI] or proportions. Unpaired T-test or Chi-squared tests were performed*.

*NA, not applicable; AHI, apnoea–hypopnoea index; TM, telemedicine; UC, usual care*.

* *The mean daily CPAP use is presented for patients still staying on follow-up visits at 3 or 12 months*.

The results of the Two-Way Mixed ANOVA showed that there was a significant main effect of time on daytime sleepiness score [F_(1.8, 123.94)_ = 53.9] and on depression score [*F*_(2, 130)_ = 11.5, *p* < 0.001]. Bonferroni *post-hoc* tests revealed similar findings for both scores, i.e., there were significant improvements between baseline and 3 months of treatment (*p* ≤ 0.001), and between baseline and 12 months of treatment (*p* < 0.001). There was no significant difference between 3 and 12 months (*p* = 0.54 for ESS, *p* = 0.99 for depression).

In contrast, there was no significant main effect of group on ESS score [*F*_(1, 68)_ = 0.002, *p* = 0.97] nor on depression [*F*_(1, 65)_ = 0.001, *p* = 0.98]. In addition, there was also no significant interaction between time and group on ESS score [*F*_(1.8, 123.9)_ = 0.5, *p* = 0.59] and on depression [*F*_(2, 130)_ = 0.1, *p* = 0.89]. Descriptive statistics are detailed in Table 3.

**Table 3:** Change in clinical variables with both interventions.

	**Time**			***p*****-value**		
	**Baseline**	**3 months**	**12 months**	**Within-subjects**	**Between subjects**	**Interaction**
				**(time)**	**(group)**	**(time × group)**
**ESS score**							
UC group	10.7 [8.7; 12.6]	5.5 [4.1; 7.0]	5.4 [4.2; 6.6]	<0.001	0.97	0.59
TM group	10.5 [8.5; 12.4]	6.1 [4.7; 7.6]	4.9 [3.7; 6.1]			
**Depression score**							
UC group	4.3 [2.8; 5.8]	2.2 [1.0; 3.3]	2.3 [0.9; 3.7]	<0.001	0.98	0.89
TM group	4.1 [2.6; 5.5]	2.2 [1.1; 3.4]	2.5 [1.1; 3.9]			

*Data are displayed as means [95% CI]. Two-way mixed analysis of variance was performed*.

*ESS, Epworth Sleepiness Scale; TM, telemedicine; UC, usual care*.

### Healthcare Time

**Figure 3:**
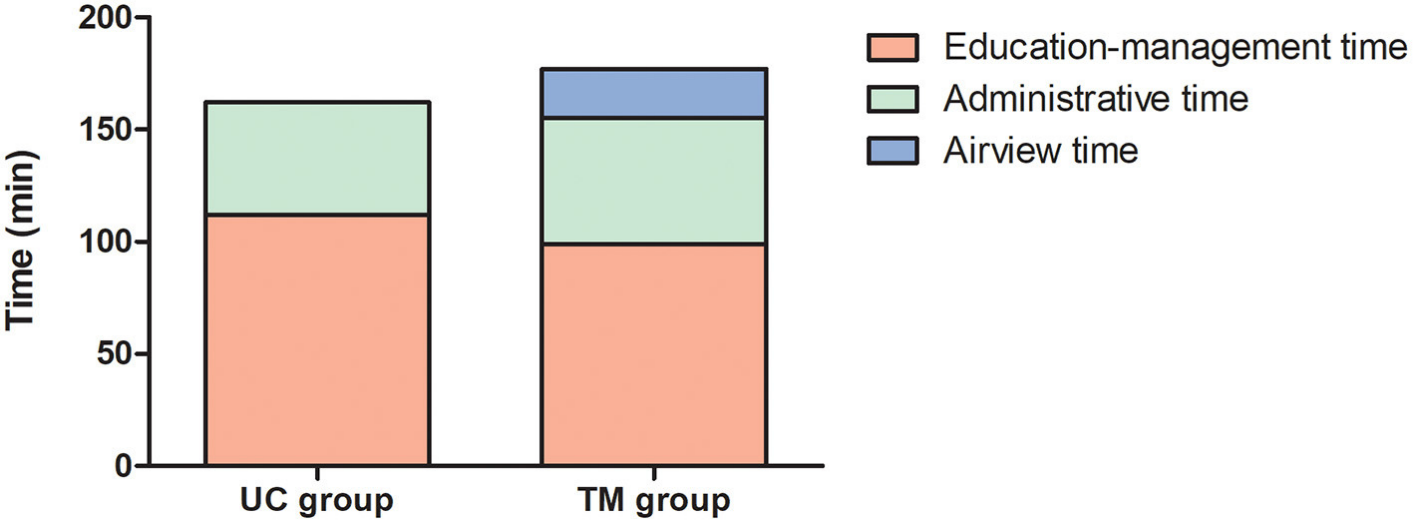
Mean healthcare time allocated in each group. Bar graphs represent the total consultation time allocated for participants randomized in the usual care (UC) and telemonitoring (TM) groups. Each bar is subdivided according to the amount of time spent for educational, administrative, or software management (Airview) purposes.

More consultations were scheduled in the TM group, yet the time allocated per consultation for each patient was lower in this group compared to the UC group (Table 2, Figure 3). Considering those both parameters together, the total amount of consultation time did not differ between groups (Table 2). In addition, the sleep care givers spent the same amount of time for administrative or educational purposes in both groups. The number of face-to-face consultations were significantly lower in the TM group compared to the UC group (Table 2).

### Costs

Values for costs estimated in both groups are summarized in Table 2. The total cost per patient was significantly lower in the TM group compared to the UC group. An average saving of 37 CHF [95% CI: (0.5–73.7)] per patient resulted from using the TM system. This difference was driven by the lower need of face-to-face visits which spared the rental of the office room and reduced the patient's displacements in the town.

## Discussion

This study showed, in a cohort of 120 patients newly diagnosed with OSA, that the usage of TM during the critical habituation phase for CPAP did not alter daily CPAP use or treatment adherence. On the meantime, TM was not associated with more healthcare resources than the usual care with standard face-to-face appointments. Therefore, TM might discharge busy clinic by supporting a parallel virtual attendance for CPAP titration, which ultimately may speed up the access to care and facilitate amelioration of care.

Two types of TM exist: synchronous, which refer to real-time clinician-patient interactions, and asynchronous which indicate that the feedback provided to the patient does not occur in real-time (23). Synchronous was the TM form used in this study, but we did not find any difference in adherence between the two arms, whether in terms of the proportion of patients discontinuing CPAP therapy or in terms of the mean CPAP utilization time. In addition, there was no counterpart on CPAP effectiveness. These results are consistent with findings from other studies. Isetta et al. randomized 139 patients newly diagnosed with OSA in two groups, comparing standard face-to-face management with CPAP televisit (video-conference consultation), and found similar CPAP compliance rates and treatment effectiveness between both groups at 6 months (24). In a randomized controlled trial comparing phone calls follow-up with standard in-person visits over 3 months, Fields et al. reached similar conclusions (25). In addition, Schoch et al. have found no significant difference in the proportion of nights with CPAP use >1 h at 6 months between the TM and control groups (26).

Overall, one of the major advantages of TM in the management of OSA consists on saving nursing time. Anttalainen et al. demonstrated that TM saved on average 19 min of nursing time compared to the usual care group, in 111 patients (18). Munafo et al. showed an average coaching time saving per patient of 25 min, in 122 patients (27). These studies used asynchronous TM [wireless telemonitoring Restraxx Online system (18) and electronic notifications *via U*-Sleep (27)], which likely explain the discrepancies with our results. Indeed, were not able to find any difference in total healthcare time spent between both forms of consultations. In our setting, teleconsultation did not imply regular transmission of data and regular check-ups. Rather, real-time teleconsultations were performed in parallel to standard in-person visits with the purpose of meeting the increasing caseload in sleep units, especially in a place where CPAP habituation phase includes three visits within 1 month and there is a long waiting for a visit. Thus, our study confirmed the feasibility of teleconsultation as a parallel route to face-to-face consultations during the critical CPAP habituation phase.

Cost-effectiveness of TM is also an advantage frequently put forward. Two recent randomized-controlled studies have shown that TM was more cost-effective than usual care (17, 24), although at the price of a lower patient satisfaction in one study (17). While we did not perform a robust cost-effectiveness calculation, we took into considerations estimates of the main costs associated with TM or face-to-face visits. In accordance with the other studies, we found slight cost savings of 37 CHF (35 EUR) per patient on average with TM, due to the reduction of travel costs and office room rentals. Yet, this calculation will likely not apply in other countries where economic times and public transportation policies strongly differ. However, our study support at least the idea that TM does not come with economic disadvantages, but the real cost-effectiveness of this technology remains to be clarified in larger series and in multiple frameworks.

Whereas, dehumanization of care with TM is a significant concern for the clinician/patient relationship, this parallel route should not be underestimated. Telemedicine has not come to completely replace in-person visits. Each form comes with its own advantages and disadvantages, and the perfect match of both routes (with and without face-to-face contacts) will likely improve medical care as a whole (28). Recently, teleconsultation has also proved very useful during the coronavirus disease 2019 outbreak in Switzerland. In this context, TM successfully fulfilled its role in improving access to care since habituation phase was maintained thanks to teleconsultation, thereby maintaining a standard of care which could not be maintained face-to-face due to social distancing rules.

Our study had also limitations. First, this study did not assess asynchronous TM, so we are not able to determine whether asynchronous care would lead to the same findings. It would be interesting to evaluate asynchronous management in order to optimize the management time. Second, this study was not designed to assess cost-effectiveness of the TM. The reduction of the costs presented is only informative only, but it would be interesting to formally investigate this aspect in a cost-effectiveness study. Finally, as 34% of patients in the UC group and 25% in the TM group had abandoned CPAP therapy by 12 months, an intention to treat analysis of the primary outcome at 12 months was not feasible and the mean daily CPAP use is therefore presented for the per protocol population still staying on follow-up visits at 12 months.

## Conclusions

After 1 year of follow-up, Telemedicine during the CPAP habituation phase did not alter the daily CPAP use or treatment adherence and did not require more healthcare time.

## Data Availability Statement

The raw data supporting the conclusions of this article will be made available by the authors, without undue reservation.

## Ethics Statement

The studies involving human participants were reviewed and approved by Communication de la Commission Cantonale d'Éthique de la Recherche (CCER) de Genève project n° 2016-01601. The patients/participants provided their written informed consent to participate in this study.

## Author Contributions

OC, SV, HT, SG, PK, and FC were involved in developing the trial concept and design. SV, AD, HT, SB, SG, PK, and FC acquired data. OC, WP, and SV performed statistical analysis. OC, WP, SV, and FC performed analysis, interpretation of data, and drafted the manuscript. OC had full access to all trial data, takes responsibility for the integrity of the data, and the accuracy of the data analysis. All authors revised the manuscript critically for important intellectual content, gave final approval of the version to be published, and read and approved the final manuscript.

## Conflict of Interest

The authors declare that the research was conducted in the absence of any commercial or financial relationships that could be construed as a potential conflict of interest.

## Abbreviations

AHI: apnoea–hypopnoea index
APAP: autoadjusting positive airway pressure
CPAP: continuous positive airway pressure
ESS: Epworth Sleepiness Scale
OSA: obstructive sleep apnea
TM: telemedicine
UC: usual care.
